# An Account of the Disease Lately Prevalent at the General Penitentiary

**Published:** 1825-07-01

**Authors:** 


### MEDICO-CHIRURGICAL

### REVIEW.

VOL. VII.] Analytical Series. [No. 21.

" Nec tibi quid liceat sed quid fecisse decebit
" Occurrat, mentemque domat respectus honesti."

VOL. III.] JULY, 1, 1825. [No. 5.

[NEW SERIES.]

### I.

*An Account of the Disease lately prevalent at the General Penitentiary.* By P. MERE LATHAM, M.D. Fellow of the Royal College of Physicians, and Physician to St Bartholomew's Hospital. Octavo, pp. 286. London, Underwoods, March, 1825.

THE subject of this volume is by no means of so local a nature as many people imagine. It embraces a great deal of etiology, pathology, and therapeutics. We purposely abstained from entering on the Penitentiary business, while only the crude mass of conflicting opinions and contradictory evidence (in the shape of Parliamentary Reports) was before us;—knowing that a more digested and instructive document was likely to emanate from one of the physicians most conversant with the facts of the case—and not wishing, for our own parts, to tread the same ground over and over again. We had another reason for delay—the very same which operated—and, we think, beneficially operated—on the mind of Dr. Latham himself.

" So long as such questions happen, from circumstances, to engage the interest of the public, or to admit the least mixture of party-spirit, medical men can hardly feel confident of their own impartiality; and they would do well, perhaps, to suspend their inquiry, and, certainly, to withhold their decision, until that interest and that spirit have had time to subside." *Preface*—ii.

It would, indeed, have been highly imprudent to come forward with a work of this kind, at a time when the editors of newspapers took the etiology of diseases into their own hands, and when coroners' juries considered themselves the proper tribunals for solving the most intricate problems in pathology! It was at this period, also, that numerous little medico-political coteries were in close divan—and when individuals gave vent to their feelings of jealousy, disappointment, ambition, and various other ugly passions, through the medium of anonymous squibs, and downright misrepresentations, in the columns of the daily prints! But now, that the hopes of some, the fears of others, and the curiosity of all, have had ample time to subside, reason and reflection may perchance serve as guides to the judgment, and philosophy may again resume that seat which party-spirit had usurped.

With the reports of committees, the depositions of witnesses, and the innumerable vague rumours which FAME, with her hundred tongues, had diffused "per mare ac terras," the public have been long satiated—and they are already consigned to the capacious womb of oblivion, with millions of other events, of far greater importance in their little day of notoriety. The documents now before us are of a very different stamp. They contain information applicable to the every day purposes of life, besides a curious and authentic account of a most perplexing endemic, which, though circumscribed within the walls of a single building in the metropolis, puzzled, and not seldom baffled the most eminent of the faculty in this empire!

It is well known that Drs. Latham and Roget were employed in the service of the Penitentiary for the period of 15 months—that is, from March, 1823, to May, 1824—during four months of which they had the able assistance of Drs. Hue, Southey, and Macmichael, to whom, especially the latter, (as will be seen farther on) Dr. L. acknowledges his obligations, particularly in certain etiological investigations that will be adverted to in the sequel.

Dr. Latham, conceiving that he should best succeed in rendering intelligible the history of the Milbank endemic, by describing the circumstances in the same order as they presented themselves to his observation, we shall endeavour to follow him, and convey to our readers a succinct exposé of the work, hoping, at the same time, that they will apply to the original for full information on each subject in detail.

The Milbank endemic did not immediately reveal its complete character. It assumed a great variety of forms, while all its symptoms did not appear at one time, or in one individual—in fact, it was only after continual observation of the disease for more than twelve months (in which period it existed and ceased, and again and again recurred in many hundred individuals) that our author could state what he now ventures to state, respecting its symptoms, nature, causes, and treatment.

The character of the disease, as it first presented itself to the notice of Drs. Latham and Roget, may be gathered from their first report to the Committee of the House of Commons, dated 5th April, 1823, of which we shall give an outline. It contains the result of one month's observations and inquiries.

The reporters state that, from the testimony of the officers, as well as the matron of the Penitentiary, the general health of the prisoners began to decline in the preceding autumn—1822. They became pale and languid—thin and feeble—unequal to their former task-work. Still there was no manifest sign of any particular disease—the number of sick received into the Infirmary not much exceeding that of former years and similar seasons. It was in the beginning of February, 1823, that Mr. Hutchison reported some marks of scurvy in a few individuals. In the last fortnight of February, nearly 50 cases of diarrhœa and dysentery were admitted—suspected to be of a peculiar kind, viz. to be "scorbutic dysentery." At this time these affections were beginning to spread widely, in different degrees, throughout the prison.

On the first of March, our author and his colleague entered on duty, and having learnt the abovementioned facts, proceeded to investigate the actual condition of the prisoners with their own eyes. They found the prevailing disease "to be the same with that which is known by the name of *sea scurvy*, and which is characterized by livid spots or blotches of the skin, especially on the lower extremities." Conjoined with the scurvy, in almost every case, there was diarrhœa or dysentery—and, in numerous cases, these last diseases existed, "where no marks of scurvy had appeared."

"But still, whether the scurvy subsisted alone, or the diarrhœa or dysentery subsisted alone, or whether they were conjoined in the same individuals, there was found in all those who suffered from either, or from both, the same constitutional derangement, denoted by a sallow countenance, an impaired digestion, diminished muscular strength, a feeble circulation, various degrees of nervous affection, as tremors, cramps, or spasms, and various degrees of mental despondency." 5.

These facts and phenomena naturally enough led to the belief that the scurvy and the bowel complaints were owing to the general constitutional derangement. They examined the bodies of two prisoners who had died dysenteric, and found, in various parts of the intestines, the morbid appearances termed ecchymoses—"that is, spots of the same kind as those which, on the skin, constitute scurvy." "We found, in fact, an absolute scurvy of the bowels, of which the diarrhœa or dysentery was only a symptom and consequence."*

The disease affected more than one half of the whole number of prisoners—but more of the women, in proportion, than the men.

"Some striking exemptions require to be noticed. Of the 24 prisoners employed in the kitchens (13 men and 11 women) belonging to the class which had suffered most extensively, all were free from the disease, excepting three, one woman and two men. These three had been promoted to the kitchen within four days. It is proper to add, that the officers and servants of the Establishment, together with their families, residing within the walls of the prison, and amounting to 106 individuals, were universally exempt from the disease." 7.

The enquiries respecting the *causes* of the disease, at this period, led the reporters to state it, as their opinion, "that the situation of the prison had not contributed to its production," an opinion which, as will be seen, they had reason afterwards to alter. We shall therefore pass over the *reasons* which the reporters assigned for their first opinion—merely observing that the circumstances and history, *at that period*, appeared to justify the conclusion to which they came, and would, in all probability, have led any other men, or body of men, to the same persuasion.

Having stated their opinion of what was *not* the cause of the disease, the reporters go on to state what they considered to be the *causes*: namely, the low scale of diet and the inclemency of the weather.

"One such cause is found, we conceive, in the diet of the prison. During the last eight months the diet was different from what it had been ever since its establishment. The change which took place in July last, reduced the animal part of the diet almost to nothing. In a soup, made of pease or barley, ox heads were boiled, in the proportion of one ox head to 100 male, and one to 120 female prisoners: and we found, upon inquiry, that the meat of one ox head weighed, upon an average, eight pounds, which, being divided among a hundred, allows only an

---

* This is an important fact for the consideration of those who maintain that *inflammation* of the mucous membrane of the intestines is the primary and proximate cause of dysentery. Here we have merely *ecchymoses*, a state or condition the very reverse of inflammation—a phenomenon which takes place very commonly *in dead bodies*, where inflammation will hardly be accused of going on. In the Penitentiary prisoners, who were walking spectres, and covered with livid blotches, dysentery destroyed life, and yet ecchymosis only was visible. This proves that inflammation is not essentially necessary for the production of dysentery, though it is a very general epiphenomenon, where the disease has continued for any time.—*Rev.*

ounce and a quarter for each prisoner. This new diet had been continued until the present time; and to it we mainly ascribe the production of the disease in question." 10.

The diet *alone*, however, was not considered the cause of the disease. The severity of the winter was looked upon as a powerful auxiliary.

In respect to the prevention and treatment, our authors ordered a change of diet, allowing the prisoners four ounces daily of animal food, and eight of rice, with other suitable addenda. The medical means need not, at present, be detailed.

Between the 12th and 19th March, the horizon of the Penitentiary cleared considerably. The prisoners worked more cheerfully—the scorbutic marks had begun to decline—and some had entirely disappeared. The diarrhœa and dysentery receded, *pari passu*, with the exterior phenomena of disease.

Between the 1st March and the 5th April, the period embraced in this report, 222 had been admitted, besides 110 whom they found in the Infirmaries. Of these, 11 had died of dysentery—and 5 of other diseases.

The report concludes with some very judicious suggestions respecting an improved scale of diet for the prisoners.

From the report, of which we have now exhibited a sketch, it is obvious, and, indeed, Dr. L. candidly confesses, that the reporters entertained no other opinion concerning the disorder than that it consisted of a diarrhœa or dysentery, and a slight scorbutic diathesis—that it arose from impoverishing diet and inclement weather—that it was already on the eve of disappearing—and, finally, that the health of the prisoners was [illegible]n to be perfectly re-established. But, alas, how shortsighted are poor mortals! and how uncertain are all medical prognostications!

"The report had hardly been made public when the disease, so far as it was referable to the bowels, began to re-appear: by the middle of the month of May it had again pervaded the prison; and by the middle of the month of June, all the prisoners, without exception, who had formerly suffered; and all, with very few exceptions, who had been exposed to its presumed causes, yet had never suffered before; and all, with very few exceptions, who had been admitted into the Penitentiary since its presumed causes had been removed, were involved in the same calamity; and the remedies, which were formerly successful in controlling it, had not now the smallest beneficial influence." 23.

It is to be remarked, however, that the scorbutic spots never returned—it was the bowel complaint that now assailed the prisoners with such violence. This brings us to the second chapter of the work, where the individual affections are taken into consideration separately, and in a more purely professional manner than could be done in a report to a parliamentary committee.

1. *Scurvy.* That condition of the surface, called "goose-skin"—a state produced by the common causes of constitutional weakness, and frequently seen among those who are ill nourished and ill cloathed, was particularly observed by the reporters among the Penitentiary prisoners, when first visited by them.

"In whatever part of the extremities this condition of the skin was most conspicuous, there were always found certain specks or spots of a blue or livid colour, formed by blood extravasated beneath the cuticle.

"In some, these spots were no larger than a pin's head, and quite circular; when they shewed themselves hardly any where, except just at the outside of the knee near the bend of the hams.

"In some, intermixed with these spots of the smaller size, were others, of the diameter of a pea, and quite circular; when they shewed themselves over various parts of the extremities, but still especially about the hams.

"In some, there were blue and livid spots of a much larger size, still preserving a sort of circular figure, and evidently formed by two or three of the smaller spots, with which they were intermixed, coalescing into one. These specks and spots of all sizes, intermingled and coalescing with each other, were distributed over every part of the extremities.

"In others, together with these specks and spots which have been described, or without them, there were large blotches of an irregular shape, and of such an extent as to leave the legs, or arms, or thighs, or buttocks, almost uniformly livid, with hardly an appearance of their natural complexion.

"In a few there was ecchymosis of the conjunctiva; and, in a few, ecchymosis and swelling of the upper eye-lid.

"These appearances, occasioned by blood extravasated beneath the cuticle, from the clusters of little livid points about the hams to the large livid patches occupying the whole limb, must be regarded as indicating different degrees of the same disease, by whatever name it is called. Some concomitant conditions, which remain to be noticed, will enable us perhaps satisfactorily to determine what that name ought to be.

"The gums were spongy, and soft, and livid, and disposed to bleed, in all those who had extensive discolourations of the skin; and in those who had mere specks and spots of ecchymosis, their condition, although it might not have been noticed under ordinary circumstances, was far from being healthy; they had a purplish hue, and were tender and sore, and often ragged, just where they come in application with the teeth. In a few, and especially in one man (Henry Peers) the mouth seemed in a state of absolute rottenness, the gums bleeding and broken down, the teeth loose, and their fangs half exposed, and the whole mucous membrane of the lips and cheeks black and ragged, while a foul cadaverous smell was emitted with the breath.

"In many who exhibited specks and spots only of ecchymosis, and in all whose limbs were covered with large blotches, the muscles of the legs were perfectly hard and rigid. In a few, the legs were œdematous, and one man (Henry Peers, already mentioned) was universally dropsical." 29.

To such phenomena we cannot but apply the term scurvy—a disease not peculiar to seamen alone, but affecting, at various times, and in various places, bodies of men on shore, as lately happened with our unfortunate countrymen, at Rangoon, in the East Indies.

2. *The Bowel Complaints.* These are represented by our author as very peculiar in their nature—being "neither a diarrhœa nor a dysentery simply," nor belonging exclusively to the bowels, "but to the whole system."

"There was every degree and species of flux that was ever seen or described. There were cases which corresponded with the descriptions of the Indian cholera. The patients were seized with intolerable cramps at the pit of the stomach. They retched and vomited, and a thin turbid serum ran from their bowels, followed by severe tenesmus. The pulse became feeble and frequent; they were pale and chilly; and a sudden anguish pervaded the whole frame. Again, there were cases which corresponded with the common autumnal cholera of this country. The patients had severe griping of the intestines generally, and cramps of the extremities, while pure and unmixed bile ran from the bowels, scalding them (as they expressed it) like melted lead in its passage.

"Moreover, there was every kind and degree of dysentery; some purged pure blood in large quantities; others, a fluid like the water in which raw flesh had been washed. In some, the evacuations otherwise healthy, were just streaked with blood; in some they contained (what seemed to be) lumps of flesh. In others they were mixed with mucus and slime, or they consisted of mucus and slime altogether.

"Again, there were cases which differed very little from the diarrhœa of common casual occurrence, except that they were quite intractable by common remedies. The evacuations were loose and attended with griping, but feculent and without any morbid quality.

"Lastly, there were cases which had no resemblance whatever, either to cholera, or dysentery, or diarrhœa, or to any disorder that has obtained a name. In the evacuations, there appeared nothing that had any sensible quality of fæces, of bile, or blood, or (of what is understood by) mucus and slime. But they consisted sometimes of a mass, like green or black grapes in a state of fermentation; sometimes of a matter like yeast; sometimes they were in colour and consistence like half-slaked lime, when it is beginning to crumble; and sometimes like a thin mixture of chalk and water, and always intolerably sour and offensive, and in enormous quantity." 34.

It is to be remarked, that the probable issue could not be prognosticated by the kind of flux only:—for those who had extreme symptoms of cholera or dysentery were as likely to recover as those who had simple diarrhœa—and those who had the *latter* were as likely to die as those who had the *former*! This being the case, it became necessary to seek for indications in the concomitant phenomena. Accordingly, they looked to the state of the abdomen, in respect to the presence or absence of pain—to the tongue, pulse, &c. The abdomen was found, in some, to be partially distended, chiefly about the epigastrium—in some, universally tympanitic—in others, retracted towards the spine—in many, the state of the abdomen was soft and natural. The pains were very variable, both in kind and in degree. Some experienced none at all, except just before or after the evacuations.

"The great majority, however had some kind of perpetual uneasiness within the abdomen. There was a very general complaint of (what was called) *sinking at the pit of the stomach*. What this sinking is, those only know who have suffered it. All patients speak of it by the same name, but do not describe it further. From observing and interrogating those who now complained of it, I suspected it to consist of a certain degree of actual pain, combined with a feeling which is akin to approaching syncope, and spreads from the stomach, as from a centre, over the whole frame. It is a painful and overpowering sensation, as if animal life itself was hurt and lessened.

"Now this sinking was not only present with the bowel complaint, but many suffered it alone, long before their bowel complaint arose; and many still suffered it long after their bowel complaint was gone. In the one case, it gave notice that the disease was approaching, before its more characteristic symptoms arrived; in the other, it was an evidence that, although its more characteristic symptoms had subsided, the disease had not actually ceased. That this painful and depressing sensation, among many other severe sufferings, was often still the greatest of all, I infer from this consideration. Patients would continually endeavour to withdraw our attention from the more tangible symptoms of their disorder for the sake of fixing it upon this. When we were interrogating them upon circumstances apparently more urgent, they would interrupt us, and exclaim 'but this sinking, this sinking; pray do something for this sinking!'" 37.

Many suffered agonizing paroxysms of pain in various parts of the abdomen, as in the regions of the stomach, bladder, loins, &c. They partook principally of the colicky character, but must, in some degree, have been owing to the diseased state of the bowels. In the great majority of cases, the tongue, during the whole course of the complaint, was quite clean, moist, and of natural colour.

The pulse, in a few cases, had frequency and strength enough to require bleeding, or depletory means short of bleeding—but, in the majority of instances, there was no morbid character in the pulse which demanded bleeding or any other kind of depletion. The same might be said of the pyrexia. Such was the remarkable diversity of symptoms accompanying this strange and multiform disorder of the bowels, the concomitant phenomena of which, instead of helping to illustrate, contributed rather to obscure the real nature of the malady! "If any form of the disorder was more formidable than another, it was that which seemed to consist in mere diarrhœa." Of this form, two cases made an early and lasting impression on the minds of the physicians. They had come, by a slow but uninterrupted progress, to a fatal issue, without any other symptom than that of simple diarrhœa. No pain—no fever. Never did the reporter witness the process of dissolution so lingering. "In the very act of dying, when the pulse could only just be felt, it had not exceeded sixty." Their purging was incessant and uncontrollable—but there was nothing particular in the evacuations, except that they were watery.

"One of these patients was examined after death, and the only traces of disease discovered, were three or four spots of ecchymosis at the upper portion of the large intestines, without any appearance of vascularity or inflammation in the neighbourhood." In process of time, cases of this kind became more and more numerous.

"Many poor wretches thus affected were given up by us as lost, and were just ready to perish at the time when we first resorted to the use of mercury. They lay in bed without fever; without pain; without excitement of the pulse; but with a turbid water continually running from their bowels. This was their only symptom; and this nothing could restrain. Their complaint (as long as they did complain) was of "that dreadful sinking." But now their complaining had ceased. Being roused, they looked up for a moment, but made no lamentation, and then laid their heads down again in despair. It was a dismal office to watch over their tardy dissolution, and witness the frustration of every expedient for their relief. These were the cases in which we first put the efficacy of mercury to successful proof; and I cannot help mentioning the relief my mind experienced from a sense of responsibility which had now become truly awful, as soon as the salutary influence of this remedy was apparent." 43.

In those who died, dissection discovered various morbid conditions in the course of the intestinal tract. They were principally of three kinds—ecchymosis—congestion of the small blood-vessels—ulceration. Many died of long-continued and uncontrollable bowel complaint, without any thing being found but a few patches of ecchymosis or of vascular congestion. In others, there were small ulcers—apparently a change from ecchymosis or vascular congestion.

"There was, however, one appearance, not unfrequently met with in our examinations, with which I was then unacquainted, and which (as far as I know) has never been particularly described. This was the appearance of ulcers in the course of their progress towards reparation. It is a question (I believe) whether ulcers of the bowels be capable of reparation at all. Here, however, there seemed to be sufficient evidence that they were so.

"In several bodies, which we examined, one, or two, or three little spots were found, corresponding, in shape and size, with the smaller ulcers, which have been noticed, where there was no remaining character of ulceration, but the mucous membrane apparently drawn and puckered, and its continuous smoothness interrupted. At these spots, closer examination, by help of a lens, discovered a circular margin, which was slightly elevated, enclosing a space which was slightly depressed. This space had a reticulated appearance, formed by minute white filaments of lymph, crossing each other in various directions, among which small red blood vessels were visible. There was no unusual vascularity of the mucous membrane in the neighbourhood, nor any alteration of its natural colour; so that these little spots would probably have escaped our notice, had we not been habitually minute in our examinations. In a few instances, however, we were led to them by observing a peculiar condition of the peritoneal coat, which seemed here and there gathered up and drawn to a point, appearing externally as if a small portion of the intestine had been taken up by the forceps and tied with a ligature on the inside. Wherever such was the condition of the peritoneum, upon examining the bowel within, we found an ulcer in (what I presume to be) the course of reparation exactly opposite to it. It is probable that, where such was the appearance of the bowel externally, the ulcer had originally extended to a considerable depth; that it had reached perhaps beyond the muscular coat, and that its reparation was in some sort necessarily effected at the expense of the peritoneum. Granulations springing from the bottom of the ulcer, as they contracted and coalesced, would pucker and draw together that part of the peritoneum from which they grew." 52.

In comparing the symptoms during life, with the appearances on dissection, "there did not appear any very strict correspondence between them." Yet there was, Dr. L. observes, a certain correspondence between them of a more general character—especially as related to the seat, though not the nature of the local affection in the bowels. "Still, upon the whole, the disease, as traced out by dissection, was far from affording an entire explanation of the disease, as manifested by symptoms during life." The following is a judicious observation, and one which we wish were more generally borne in mind by those pathologists who trace certain constitutional disease (as fever) to a local origin.

"But the entire disease does not always consist in its visible marks upon particular organs. If injury be done to a healthy body, there, indeed, it may; and its anatomical character simply may become the best criterion, whether it be of easy or difficult reparation. But, where a visible change of structure arises, independent of injury from without, there must be something within the body that preceded, and conduced to it. This something, this inceptive movement, whether it be of the part or of the constitution, which foreruns the actual manifestation of visible disease, will not bear to be spoken of with precision. We talk of cachexies, of constitutional taints, and morbid dispositions, not knowing how to define what we mean. This, however, we know, that the local diseases which follow the conditions we thus designate, upon whatever part of the body they fall, are much more difficult of cure than their mere anatomical character would imply." 56.

It is certain, observes Dr. L. that, at the Penitentiary, long before the manifestation of any particular disease had appeared, the general health of the prisoners had begun to decline. Thus, although scurvy and bowel complaint did not appear till February, 1823, the prisoners had become "pale, languid, thin, and feeble," according to the testimony of the officers, in the preceding autumn.

This universal cachexy, although unknown as to its precise nature, was something real in fact—and was perhaps the most important part of the endemic.

*Treatment.* In the beginning of April the flux, which affected between four and five hundred people, had given way almost entirely to chalk mixture and laudanum, aided by an improved scale of diet. But when the bowel complaint returned at the end of that month, it was no longer amenable to the same remedies. Still they endeavoured to reach the disease, by combating conspicuous symptoms. For pain, aggravated by pressure, and attended with pyrexia, blisters, fomentation, bleeding, &c. were employed, and so on with other symptoms; "but the consequence was only a brief respite from suffering while the flux continued." Then were employed the train of astringents, bitters, aromatics, antimonials, ipecacuan, &c. but all in vain. The anceps remedium, that much *abused* (in every sense of the word) medicine, MERCURY, was yet in reserve; but "owing to considerations which had an unavoidable influence upon our minds, some time elapsed before we resorted to its employment." They understood, indeed, that a fair trial had been given to the remedy before they took charge of the Penitentiary, which was one reason of delay. Another was, that scurvy had been combined with the flux;—an adjunct which, upon medical theories (the bases of which are well known to be as immutable as the sun) was an insuperable objection to the use of mercury. The extract we are now about to introduce is long, but indispensible.

"While two hundred individuals were suffering a flux of the bowels at the same time, and many seemed gradually approaching to their dissolution; and while the numbers of the sick were every day increasing, and the forms of the disease were becoming more and more various and complex, and all the methods of treatment hitherto employed had served to palliate only, and not to cure, Dr. Roget and myself determined, after mature deliberation, to get rid of all restriction upon our practice, which had arisen from the consideration that the flux was originally combined with scurvy; and we agreed to employ mercury, in such forms and combinations as the exigencies of particular cases might seem to require.

"We first made trial of this remedy in those cases which our experience had brought us to regard with the greatest apprehension, cases (if I may so say) of mere passive diarrhœa, where there was no excitement of the circulation, where there was little pain, and little of morbid quality in the evacuations, but where the evacuations were enormously frequent, and hitherto absolutely incontrollable. In these cases all medical expedients had failed, and we were now compelled to content ourselves with such temporary relief, and such short intervals of ease, as opium, administered in draughts, or clysters, or in cataplasms, was able to procure. In our trial of mercury for these cases, we proceeded thus:—Equal quantities of hydrargyri c. cretâ and pulv. ipec. comp. were made into pills; each pill consisted of five grains, two grains and a half of each ingredient, and one of them was administered, three times a day, to about twenty patients. Still there was no abatement of the diarrhœa. They were administered four times a day, and still the diarrhœa continued. They were given five times a day; when, upon our next visit to the Penitentiary, we found, among those who had taken mercury, one female in a profuse salivation, and the diarrhæa completely arrested in her, and in her alone. This poor creature had formerly had scorbutic spots upon the skin, at the same time that she suffered a flux of the bowels. The scorbutic spots had disappeared altogether; the flux had subsided, but returned; and that form of it, which has been described, had now brought her life into imminent hazard.

"In this instance, the salutary effect of mercury was unquestionable; and the condition of its success seemed to be, that it had procured salivation. We proceeded, therefore, more boldly in the use of it, still giving it in the same form, and in combination with Dover's powder. We increased the dose to those who already took it; and, as they became salivated in succession, they were all freed from the symptoms of their disorder. We subjected more and more of the prisoners to the same treatment, watching them carefully in the mean time, for the sake of still more confidently ascertaining the precise condition which was essential to the success of the remedy. This we uniformly found to be the production of salivation.

"Be it remembered, that these cases, upon which the salutary effect of mercury was first proved, were those which occasioned us the greatest apprehension. Several of the patients were so feeble and emaciated, so pale and faded in their aspects, that while, on the one hand, we were feeling our way with the mildest preparation of mercury for the purpose of curing their disease, we were, on the other hand, administering wine and cordials for the purpose of upholding their existence.

"The success of mercury, under these unpromising circumstances, led first to the more general, and, finally, to the universal employment of it: We resorted to it in every case of flux, where the remedies hitherto used had not satisfied our expectation. In short, we resorted to it in every case without exception.

"But, as it became more and more obvious that salivation was the condition of its success, there was no reason for restricting its use to one preparation only. Yet, at the same time, it was not enough that salivation should be procured in any way, gradually or at once, quickly or slowly.

"Experience taught us, that its curative effect depended, in some degree, upon the manner in which salivation was brought about. Hence, a choice and a discretion were to be exercised upon the kind of preparation, the quantity and frequency of the dose, and its combination with other remedies.

"Where the flux was attended with severe tormina, or colic, or cramps at the stomach; or where the attack was sudden, and recent, and accompanied with fever, it was doing nothing to prescribe small doses of hydrarg. c. cretâ and Dover's powder, which would produce their effect some days hence. It was expedient that the impression of the remedy should be in proportion to the force of the disease, and the rate of its progress. Accordingly, large doses of calomel and opium were given, to make the mouth sore immediately, or as soon as it could be done with safety.

"In several cases, in which the agony from tormina and tenesmus was extreme, and the evacuations were enormously frequent, and consisted altogether of morbid secretions, or blood, fifteen grains of calomel and two grains of opium were given at a dose.

"The patients, to whom so large a dose of calomel was given, were most attentively watched. Especial care was taken that nothing should divert it from its influence upon the constitution, and that every accidental inconvenience that might accompany its operation should be rendered as tolerable as possible. If the griping increased, they had peppermint water to have recourse to; if it still increased, they were to be largely and frequently fomented with flannels wrung out of warm water; and if it still increased, they were to be supplied with small doses of laudanum at short intervals.

"It will hardly be expected, that this single dose of calomel and opium could be effectual to the complete cure of the disease. The degree of relief which the patient experienced the next day, and the changes which his condition had undergone in the mean time, determined the manner of proceeding in the further treatment of the case.

"The dose of fifteen grains of calomel and two grains of opium, administered under such emergencies as have been described, had almost always the effect of calming the symptoms; but the degree of relief it procured was various.

"On the next day, we sometimes found that the patient had past an easier night, that the evacuations had been somewhat less frequent, and the tormina and tenesmus had been somewhat moderated, but that, since the morning, the symptoms had become worse again, the pains were as severe as ever, and the evacuations as frequent, and quite unaltered in their appearance. Under these circumstances, fifteen grains of calomel and two of opium were given a second time.

"Sometimes, the day after the first large dose of calomel and opium, we found the relief, which had been procured through the night, still maintained, and the appearance of the evacuations changed, in some obvious respect, for the better. Perhaps they were now free from all admixture of blood, which they contained the day before. Under these circumstances, half the former dose of calomel and opium was given.

"Sometimes, the day after the first large dose of calomel and opium, we found the patient exulting that he had been cured as by a charm; that he had slept all night, and his pains were gone; and that he had had several evacuations, of which the two or three last were almost natural. With this sudden improvement, salivation had either already arisen, or it was at hand. Under these circumstances, the use of mercury was either suspended altogether, or small doses of calomel and opium were given until ptyalism appeared, which was generally obvious at our next visit.

"Our ultimate object, in all cases, was to produce salivation. But, in these cases of severer suffering, we found a salutary impression capable of being immediately produced by a few large doses, or even by one large dose of calomel and opium. This it was expedient to make the most of. Nevertheless, this immediate salutary impression was soon lost, unless the same practice was followed up to salivation; for which purpose mercury was afterwards sparingly or largely exhibited, according to the circumstances, which have been set forth." 73.

We think the foregoing document is a tolerable set off against the clamour which has lately been raised against the use of mercury in the bowel complaints of hot climates. This clamour we well know to be founded almost entirely in prejudice—or, what we are sorry to say is worse than prejudice—in the personal opposition of writers. But, "omnia vincit veritas, et prevalebit."

In many, the constitutions were most reluctant in admitting the specific action of mercury—and the disease continued its ravages till, by the aid of mercurial frictions, ptyalism was produced, and then the usual relief followed.

In many, the process of cure was so gradual, that there was hardly any perceptible *action* engaged in it. "The constitution seemed rather to lose the disease, one symptom after another, than to surmount it by an effort of its own." In others, the process of cure was by a sudden, vigorous, and painful effort, when the constitution threw off its disease by a sort of critical paroxysm. "Sometimes the critical effort commenced as soon as the mercurial fœtor was perceptible in the mouth. Sometimes salivation would exist 24 hours before the crisis began—and sometimes the crisis preceded the salivation 24 hours. But it never took place except where there was salivation at the time, or immediately before or immediately after." 74.

"The critical effort was of this kind. After a calm, procured by one or two large doses of calomel and opium, or after the employment of inunction for two or three days, the constitution would become suddenly roused, and a very severe griping would arise, and then a sensation would follow, as if the bowels were filling and distending themselves with something, and, afterwards, an incontrollable urgency to stool. With the evacuation came the relief of all the preceding misery. The stools were entirely changed. A few hours before they consisted, perhaps, of slime or blood, or some colourless turbid fluid. Now they were a colluvies of the foulest, blackest matter, and of every kind; heavy, ropy mucus and bile formed a considerable part of them. After one or two such evacuations the patient felt himself entirely restored and well. It generally happened, however, that the same sort of paroxysm returned, and was terminated by the same kind of relief. Thus, after a whole night spent in a succession of these critical paroxysms, the patients were found, the next day, bathed in a warm perspiration, and fast asleep; and, from this time, the evacuations from the bowels became natural and healthy." 75.

Many a time have we witnessed these workings of the constitution in the same disease, and aided by the same remedy. More than once or twice have we felt them personally.

It has often been urged that, however successful this practice may be in the hotter regions of the earth, it is totally inapplicable to this climate—as if men entirely changed their nature by doubling the Cape of Good Hope! The Penitentiary endemic has proved the fallacy of this position—and in so doing, it will save the lives of thousands in these Islands, as well as in those vast regions—

"Where Andes, giant of the Western Star,
"Looks from his throne of clouds o'er half the world."

3. *Disorders of the Brain and Nervous System.*—The prominent phenomenon of the Penitentiary endemic—the bowel-complaint—was yet only one of its features. It has been described first, in order, by Dr. L. because it was among the first in order of time, as respects his attendance. It was only gradually that the physicians discovered the entire character of the endemic, and found that it was neither a dysentery nor a diarrhœa, *simply*—nor belonging exclusively to the bowels—but pervading other organs and systems, and, in fact, belonging to the whole constitution. 'No part of the disease, he observes, was more striking and characteristic—none more formidable than that which declared itself through the medium of the brain and nervous system.

In the report of the 5th April, the physicians alluded, among the symptoms of constitutional derangement in the prisoners, to "*various degrees of nervous affection, as tremors, cramps, spasms, and various degrees of mental despondency.*" These were attributed, at that time, to constitutional debility, and not considered as essentially connected with the predominant disease. In process of time, however, the disorders of the brain and nervous system became more and more frequent, and of various kinds, as head-ache, vertigo, cramps, twitchings of the limbs, delirium, convulsions, and apoplexy. They had not been in attendance on the Penitentiary more than a week, when a man, 31 years of age, suffering from cramp and diarrhœa, died suddenly apoplectic. On dissection, they found the vessels of the brain slightly turgid, and a few spots of ecchymosis on the intestines, although there had been no scorbutic spots on the skin. About ten days afterwards, a woman, 26 years of age, who had been suffering from head-ache, became suddenly maniacal—and she, also, died. Upon dissection, no morbid appearance could be detected beyond a very slight congestion of the blood-vessels of the brain—a congestion so slight, that its very existence might be questioned. Soon after this, they one day observed a young woman suffering most agonising spasms, her legs and arms being as tense as in tetanus.

"Suddenly she gave a loud shriek, and her eyes were fixed, and she became as pale as death. No pulse could be felt, and her breathing was only just perceptible. By æther and ammonia, and all the means of stimulating within our reach, we succeeded in rescuing her. In two or three minutes perhaps (but it is not easy to reckon time on such occasions), we could feel the pulse beginning to undulate, and see the countenance beginning to redden, so that it was evident that the blood was in motion again. Then her eyes began to pass from object to object, and it was plain her consciousness had returned. She could not yet speak; but, by inarticulate sounds, and by the motion of her hands around the heart and stomach, she made us understand, that it was there the sudden agony had seized upon her. This young woman survived six weeks; and, in the mean time, all her dreadful symptoms frequently recurred, and her existence was upheld, from hour to hour, by the most potent stimulants. If she was left more than a certain period, without a small quantity of brandy, her pulse became imperceptible; but it was felt again, as soon as the stimulus was again applied. And, in this manner, even for as long a time as six weeks, existence was still maintained, while it was continually tottering upon the verge of dissolution. Before her death, diarrhœa was added to her other complaints. Upon dissection, nothing was found at the origin of the nerves, to account for the dreadful symptoms referable to them. At intervals, throughout the intestines, there were small circumscribed patches of red, occasioned by blood accumulated in the small blood vessels; but there was no ecchymosis." 83.

These cases occurred in the month of March, and began to excite suspicions in the minds of the physicians, that they had a complicated disease to combat. Early in the month of April, the scurvy and bowel complaints nearly disappeared. The bowel complaint, as was before observed, returned again before the end of April, in an aggravated form, and with it appeared nervous affections of every kind—but of that kind especially, which betrayed itself in cramps of the muscles. These complaints occurred both in those who had, and in those who had not the bowel-affections—in men and in women—but in the *latter* with much greater severity than in the former. Many women were affected in the way the unhappy female was, whose case is detailed above—and the physicians were apprehensive that they would all come to the same wretched end. They had cramps in the limbs and in the trunk. A few of them had that indescribable agony at the heart and pit of the stomach, bringing with it those frightful circumstances which seemed to threaten instant dissolution. They were often in the greatest danger, but only one of them died. This was a young woman, aged 23 years, convalescent from scurvy and diarrhœa, in whom arose a succession of complaints in every part of the body, and with them that frightful agony at the pit of the stomach—an evident spasm, attended with severe pain. She lingered like the patient already mentioned—and her life was upheld by brandy and laudanum for some time. A few days previous to death there appeared a remarkable lividity upon the extreme parts of the body. Dissection shewed only some small vascular patches on the mucous membrane of the intestines. In these and in many other cases, there was every reason to believe that spasm actually affected the heart itself. In one instance, without any previous spasms of the extremities, or pains in the chest, and without any warning symptoms, the functions of the heart were suddenly suspended, and the patient died. He was a young man, aged 29, who had been a good while affected with scurvy and diarrhœa, but not in a state that at all portended the approach of death. On the 24th June, while the physicians were speaking to him, and while he declared himself better, his pulse became exceedingly slow, and in a few minutes ceased altogether. He was apparently dead: —but by various measures of resuscitation he was revived for that day. The next day terminated his sufferings, having been dreadfully convulsed in the interval. On dissection, little vascular patches were found on the mucous membrane of the intestines, and some increase of vascularity in the brain, with slight serous effusion there. The heart was free from disease.

There were several cases of phrenitis. To sudden and acute pain in the head were added vertigo, confusion of intellect, twitching of the tendons, strabismus, dilated pupils—and lastly, distortion of the mouth and hemiplegia. The pathologic condition was obvious; but not so the *methodus medendi*. For in almost every case of this kind, the pulse was extremely feeble. Here then was the disease, without that force of the circulation which is deemed essential to maintain it, and which is considered a necessary indication for the treatment! This caused great perplexity. Bleeding was cautiously ventured on, but no good. Blistering sometimes gave temporary relief—but some lives were lost before the effectual remedy was found out.

"Thus far the flux of the bowels, and the disorders of the brain and nervous system have been spoken of separately, as if they were essentially distinct, and their occasional occurrence in the same individuals was merely accidental. In process of time, however, a belief arose of some natural alliance subsisting between them. As soon as the disorder of the brain and nervous system became co-extensive with that of the bowels, it had an equal claim to be regarded as *the disorder* of the Penitentiary; and, as soon as both were not only co-extensive throughout the prison, but co-existent in the same individuals, almost constantly and inseparably, insomuch, that hardly an instance occurred of one being present without the other; and, moreover, as soon as the sole remedy of the one was found to be the sole remedy of the other, there seemed to be enough to constitute the requisite proof, that they were, in a certain sense (and that the most important sense,) one and the same disorder.

"Disorders may be different in the manner of declaring themselves, and may go by different names, according to the organs which they involve; and yet they may have in common one morbid condition of the system at large, from which they are derived, rendering them the same in their origin, and requiring them to be treated by the same remedy.

"Thus does Nature often bring together what nosologies and artificial arrangements have put farthest asunder; and thus does it become possible, that convulsions and dysentery, although in their symptoms they are absolutely unlike, may be essentially the same, and curable by one and the same remedy.

"It remains to speak of the various disorders of the brain and nervous system, and of the various kinds of flux, as they were found in combination. For the manner of their alliance in the same individuals, and certain conditions which they possessed in common, seemed especially to lead to the belief that they were, in the sense which has been intimated, the same disease.

"One condition, common to both, was the frequent suddenness of their accession; and there was an alarm connected with it which rendered it the more observable. Those who, to-day, were quite free from pain and disorder of the bowels, and appeared with the aspect, and with the perfect consciousness of health, to-morrow we found purging blood, or pure bile, or a turbid, watery fluid, and completely beat down and mastered by their disease; and, in like manner, those who, to-day, had the same appearance and consciousness of health, and were alert and cheerful, and working at their trades, to-morrow we found so giddy, that they could hardly stand, or confined to bed with racking pains in the head, with twitching of the tendons, or cramps of the muscles, or even with delirium.

"Here was an instant transition from the midst of health to the midst of disease in both.

"But where the accession of the disease, either as it belonged to the brain and nervous system, or to the bowels, was not thus instantaneous, still there was often a condition common to both in the character of their premonitory symptoms.

"There was a pale, and faded, and melancholy aspect, which, in process of time, had become so familiar to us, as the harbinger or attendant of the disease in all its forms, that we were accustomed to select from among the prisoners those in whom it was most conspicuous, and send them into the infirmaries, for the sake of having them more constantly under our observation. Wherever this well-known aspect appeared, it was certain that the symptoms of real disease would soon arise; it might be from the bowels, or it might be from the brain and nervous system; and, in fact, it was as often from one as the other.

"As to the alliance of these diseases in the same individuals, no form of nervous complaint was more common than head-ach and vertigo, and they were often combined with the bowel complaint in the following manner:

"The attack was introduced by simple head-ach or vertigo, the bowels yet remaining in a perfectly natural state. Some of the patients thus affected, when the head-ach and vertigo were not urgent, were simply brought into the infirmary and watched; all medical treatment being postponed for the present. Others, in whom they were more severe, were bled with leeches, or blistered, or had such *common* remedies administered to them as they seemed to require. But every patient, almost to a man, some the next day, some (the greater number) in two or three days, and some at a more distant period, were overtaken by some species of flux. The flux followed, whether the head was relieved or not, and when the flux was established, the head-ach or vertigo continued or ceased indifferently." 98.

The appearances on dissection have been anticipated. What they found, when they found any thing, was some degree of vascular fulness of the brain and its membranes, and some watery effusion between the membranes, and into the ventricles. Both were generally inconsiderable. There were cases where death took place by an immediate interruption of the cerebral functions, and yet the brain and its membranes were found perfectly natural on dissection. It has already been seen that common remedies had little or no influence on this formidable disease of the brain and nervous system. The physicians were therefore obliged to look to others of a more powerful nature, though untried in this class of complaints.

"And thus it happened with the few cases of this kind at the Penitentiary; they passed, uninterruptedly, to their fatal termination, their symptoms hardly receiving the smallest check or abatement from the remedies employed.

"Now, before we resorted to the use of mercury for the various forms of the disease prevalent at the Penitentiary, the state of the prison, in regard to that form of it which involved the brain and nervous system, was this:—Seven had already perished under our own observation; of whom one died apopletic, one maniacal, two with the symptoms of phrenitis, two from cramps referable to the region of the stomach and the heart, and one from symptoms belonging in part to the heart and in part to the brain; and there were not less than two hundred now labouring under various degrees of disorder belonging to the same organs. Of them a few only were dangerously ill in respect of the magnitude of their symptoms. These few were suffering that insidious form of phrenitis already described, while we were checking the symptoms and postponing the progress of their disease, with little hope of eventually saving their lives; and, unquestionably, they were in great present peril. But all the rest, though not in imminent danger from the magnitude of their symptoms, gave just cause for anxiety, from the consideration that they had not been cured by any means hitherto employed, and moreover that they were under the same conditions of disease through which the seven had passed, before they reached their fatal consummation.

"Such was the state of the prison in respect to the disorders in question; and it may well be conceived that, under great apprehension for the event of all these cases, and under great present alarm for a few, we sought most anxiously for the means of their more successful treatment.

"At this time many patients, in whom mercury was first successfully employed for the cure of diarrhœa, were likewise freed from certain obscure nervous complaints; some from head-achs, and some from vertiginous sensations. This occurrence, while it served to strengthen the belief that the flux of the bowels, and the nervous affections, had a natural alliance, and were, in some sort, the same disease, determined us to give the same remedy a fair trial in its application to both.

"And first, we most eagerly resorted to its use in those cases which occasioned us the greatest present alarm, *viz.* in three cases of insidious phrenitis. The patients already suffered subsultus of the tendons, and delirium, and one had strabismus. If life was to be rescued, it could only be by giving the remedy in such a manner as to bring the constitution as speedily as possible under its influence. Accordingly, as much calomel was prescribed, in repeated doses, and in combination with opium, as procured salivation in thirty hours; whereupon the most formidable symptoms were at once dissipated, and the patients were left in a condition favourable to recovery; and they eventually did recover.

"Next we resorted to its use in certain cases which occasioned us peculiar perplexity, and some apprehension of distant consequences, but no present alarm. There were many individuals in whom an affection of the head had been originally combined with bowel complaint. The bowel complaint had been very slight, and of short duration, and had ceased altogether during many weeks. But the affection of the head had been very severe; and, although it had obtained a few respites from common remedies, it still remained unmitigated; and at length all our medical expedients had lost the little temporary influence which they once possessed. There was every motive for trying the effect of mercury in these cases, and it was tried, and succeeded.

"One of these cases was peculiarly striking, and is well worth relating as an example. Upon our first visit to the Penitentiary, on the 1st of March, we found, among the patients in the infirmaries, a young man of the name of Robson. He was twenty-one years of age; and told us that he had suffered a head-ach of the most excruciating kind during several months. His sight was dim, and he had a constant twinkling of the eyelids, and great agony was depicted in his countenance. He had moreover that faded, pale, and melancholy aspect already alluded to. Yet the functions of his bowels were performed naturally. His tongue was clean, and his pulse was of the natural frequency and strength. At this time the alliance between the disorders of the nervous system and the bowels was hardly ascertained; therefore no inquiry was made whether he had suffered diarrhœa. Subsequently, however, we learnt that, during the preceding winter, his bowels had been twice slightly disordered for a few days. This poor fellow was under our constant observation and treatment, from the beginning of March to the end of June. He gained no respite from his agony, but by means of leeches applied to the forehead, day after day, for a week together, or by blisters kept open, or applied in quick succession behind the ears, or on the nape of the neck; and the respite thus obtained was of short duration: it might be for ten days; it was never longer than a fortnight; and then the same agony returned, and the same cruel treatment was to be resumed. In this case we eagerly resorted to the use of mercury; salivation was procured and maintained, to a certain degree, during several weeks. Whereupon the patient was released from his misery, and ever afterwards, during eleven months that I had the opportunity of watching him, he continued free from complaint of every kind.

"The beneficial influence of mercury upon that part of the disorder of the Penitentiary, which belonged to the brain and nervous system, soon became as unquestionable as upon that which belonged to the bowels. It was proved upon the various forms, both of one and the other, and most conspicuously upon that form of each which occasioned us the greatest present alarm.

"Before the use of mercury, it was impossible to contemplate the state of the prison, and not consider an extensive mortality as inevitable. Experience of its effects during a fortnight entirely changed our anticipations of the result, and encouraged a hope that, with great care and vigilance in its administration, the mortality would still be kept within narrow bounds. Thus, for every species of nervous complaint, as for every species of flux, whether they were combined or separate (for either might occur alone, although they were generally found together), we were led by our own experience to the employment of mercury.

"Seeing that the head-ach and vertigo were often the first symptoms, and that they often subsisted a considerable time alone before the accession of others, we thought it expedient to begin the treatment of the disease, as soon as it shewed itself under this form, by our most efficient remedy. Our experience hitherto had been, that these affections of the head, when they were the first to declare themselves, were very seldom controlled, or in any way relieved by common remedies, and that, whether they were relieved or no, the flux almost inevitably followed. Treated with mercury, however, in the great majority of instances, they ceased; and, where they did cease under such treatment, in the great majority of cases, no flux followed.

"Thus did this remedy effect the relief of both disorders, when they appeared in combination, and of each, when either occurred alone; and, in the latter case, under such circumstances occasionally, that it seemed to prevent the accession of the other." 112.

We could not abridge the foregoing most interesting and important document, without greatly lessening its effect upon the professional mind. We therefore gave it entire.

In the administration of mercury for the relief of the complaint in question, the physicians observed that, in the great majority of cases, no striking abatement of symptoms took place until salivation was procured. The abatement of symptoms, however, did not appear to be dependant on any "*certain degree* of salivation." They were, therefore, content with any, the least grade of that effect of mercury under which the symptoms would disappear. As in the bowel complaints, so in the affections of the brain and nervous system, the condition most essential to the success of the remedy was this—"that the force and rate of its impression, should be in proportion to the force and rate of the disease." Their chief object was, therefore, to preserve that proportion.

"Thus, where the disease was less severe, and was slow in its progress, salivation (without reference to its degree) was to be procured gradually; where the disease was more severe and rapid in its progress, salivation (without reference to its degree) was to be procured at once. Head-ach and vertigo which had come on tardily, and had abided many weeks, without any perceptible excitement of the circulation, were to be made to yield under the slow and alterative influence of mercury, which the constitution could bear without injury. Head-ach and vertigo which had been sudden in their accession, were accompanied with excitement of the circulation, and already seemed to threaten something beyond themselves, as convulsion, or delirium, or phrensy, were to be at once mastered by such a sudden and powerful impression of the remedy as the constitution would severely feel. Hence the quantity of the remedy was continually varied, according to the exigencies of particular cases. For some we prescribed one grain or two grains of calomel, with a small quantity of opium, once or twice in twenty-four hours, and thus succeeded in procuring relief after the lapse of a week or ten days; doing no harm, in the mean time, to the general health and sensations of the patient. For others, we prescribed five, or ten, or even twenty, grains of calomel, with proportionate quantities of opium, once, or even twice in twenty-four hours; and thus succeeded in dissipating the symptoms at once, and in rescuing life at the expense of some present injury to the constitution." 115.

4. *The Fever*. Both in the fluxes and diseases of the brain and nervous system, the fever was so very moderate, or rather equivocal, as to raise a doubt whether it was derived from the local complaint, or the local complaint from it. For Dr. Latham's own part, however, he believed that a fever arose at this time in the prison, which was *sui generis* and idiopathic, although its character might be obscured by an association with the forms of disease already described.

"When this fever occurred alone (as it sometimes did, even at the time when the bowel complaint, and the disorders of the brain and nervous system were most prevalent), its type was manifestly peculiar. It was a fever of very moderate excitement, and generally went off in three or four days by perspiration. Or, if it failed of such relief, either spontaneously or by the help of medicine, it was apt to be protracted in the form of hectic during several weeks. When this fever occurred (as it generally did) in combination with some form of bowel complaint, or some affection of the brain or nervous system, its own peculiar type was still visible, notwithstanding certain differences which it exhibited correspondent with the disorder of a particular organ." 117.

While the flux and disorders of the brain and nervous system prevailed to their largest extent, the cases of fever were rare. It was when these complaints began to subside, that the fever shewed itself sufficiently to enable the physicians to become accurately acquainted with its type. Although it never prevailed to the extent which the other complaints attained—yet it was such as to lay just claim to be considered as a part of *the disease* of the Penitentiary—springing from the same cause or causes which occasioned the others. Here our able author gives an excellent graphic delineation of the fever, both alone, and in its combinations, which we strongly recommend, as, indeed, every part of the work, to the attentive perusal of the reader; since, from the great space which we have dedicated to extract already, we can devote but little to this part of the subject. We shall quote a single case as illustrative of the fever in its greatest pitch of severity.

"A young woman (Mary Chapman), aged twenty-six, was attacked, like the rest, with the common symptoms of fever, except that she had a shivering fit, which was remarkably severe. After which, there arose a sudden and excruciating pain in every part of the abdomen, a vomiting of bilious matter, and a profuse purging of matter like tar. The patient sank at once into a state, from which it was evident that she could never rally. Her countenance was pale and full of terror; and, if she was moved, she was ready to faint away. After the lapse of twelve hours her pulse was imperceptible, and she was thought to be dying. Nevertheless, she survived four days; and, in the mean time, nothing could be attempted but to uphold life by such small quantities of wine or brandy as she could take. On the third day her constitution in a manner re-acted, and her countenance was a little flushed. Soon afterwards her respiration became stertorous, and her mouth was a little distorted, and then she died.

"After the first gush of matter from the bowels, no further evacuations took place spontaneously, or could be procured by medicine. There was no tension of the abdomen, but, on the contrary, it was flat and soft to the last. Upon opening the cavity of the abdomen we found, here and there, throughout the whole course of the intestines, from the stomach to the rectum, large and extensive vascular patches, all of a very dark colour, and some absolutely black. The great end of the stomach, and the whole of the duodenum, were intensely black. The small intestines were, in several places, puckered up and contracted, for the space of an inch; and wherever these contractions were found, the bowel was of a deep black colour. Within the cavity of the abdomen, a small quantity of fluid blood (about two ounces) was found. It seemed to have exuded from the surface of the duodenum, just where its last turn commences, and especially from that part of it which is uncovered by peritoneum. It was just in this situation that the blood was found, and this part of the intestine was soaked in it. There was no effusion of lymph, or of any other fluid within the peritoneum, but the blood above-mentioned.

"The stomach, being opened, was found empty; and at its great end, the mucous membrane seemed in one uniform state of ecchymosis. But this was not really the case; for when the stomach was held up to the light for inspection, it was evident that no extravasation had taken place, and that the apparent ecchymosis was occasioned by every vessel, great and small, in this part of the organ, being filled and gorged with blood. There was also this same appearance of ecchymosis in the mucous membrane of the duodenum, occasioned by a condition of the blood vessels essentially the same. Upon the valve of the pylorus, however, there were three small spots, where blood was actually extravasated. At the various spaces of the small intestines, which were black from without and contracted, there was the same apparent ecchymosis of the mucous membrane, which was, in fact, a remora and accumulation of blood within the blood vessels. The whole tract of the intestines was filled with a matter resembling tar.

"In the whole course of the bowels there was only one small ulcer. This was in a part of the small intestines, most free from vascularity, and seemed to be undergoing a process of reparation.

"The vessels of the brain and its membranes were loaded with blood. But there was no effusion of fluid any where within the cranium." 124.

Other cases are related illustrative of determinations to the head or other important organs, and for which we must refer to the work itself.

In these severer forms of the Penitentiary fever, as soon as there was a spontaneous gush of morbid secretion, upwards and downwards, then the cramp at the pit of the stomach or the distressing head-ache ceased—the pulse rose—and a universal warm perspiration broke out. These phenomena naturally led the physicians to imitate the efforts of the constitution, and direct their remedies towards the same channels which it employed for its own relief. Purgatives simply did not do in these cases. By them, indeed, the evacuations were increased in quantity, but not improved in quality. If the means calculated to soothe pain and quiet the bowels were first successfully employed, then the administration of purgatives had the desired effect. Thus, leeches were first applied to the pit of the stomach, succeeded by warm fomentations to the whole of the abdomen—then two or three grains of calomel were given every hour or second hour, *ad vicem secundam vel tertiam*—lastly, a drachm of the sulphate of magnesia was given every hour until the bowels were moved, when these means seldom failed to bring away copious evacuations of a foul and dark-coloured colluvies, dissipating the severe epigastric pain and distracting head-ache, and, with them, all the danger of the disease. In this place, Dr. Latham has added a note, indulging (which he rarely does) in some theory respecting the probable pathology of the cases under consideration. We think there is much sound sense and good reasoning in this said note, and, therefore, shall quote it here.

"These cases were so peculiar in themselves, and the mode of relief, whether spontaneous or by the help of medicine, was so striking, that I may be permitted (in a note, at least) to make a few observations upon their probable pathology.

"Formerly much stress was laid upon 'turgid matter' and 'morbid colluvies' in the first passages, as a cause of fevers; and certain distinctions of symptoms were thought to indicate that this colluvies was 'pituitous' in one case, and 'bilious' in another. At present, medical men are content to speak in more general terms of an accumulation of 'morbid secretions,' still regarding them as the cause of disease, and directing remedies for their removal. Emetics are given, and foul matter is rejected by vomiting. Purgatives are given, and the same is passed by stool. In consequence of which, the previous symptoms cease, or are greatly mitigated; and thus the theory is confirmed.

"Nevertheless it may still be doubted whether the popular notion be correct respecting the actual condition of the stomach, at the time the symptoms referred to it are most intense; and still more, whether the matter rejected be really the 'materies morbi,' the accumulation of which produced the symptoms, which cease upon its evacuation.

"It consists better with sound pathology to believe that, at the period of the severest local pain and severest constitutional disturbance, there is hitherto no accumulation of morbid matter within the stomach, but that its blood-vessels are engaged in a morbid process, which if its termination is favourable, will be finally resolved by a gush of foul secretion from their extremities.

"That it is not any thing extraneous to the blood vessels which produces the symptoms, but the blood vessels themselves by their own morbid action, is rendered probable by the relief which leeches often procure, when they are applied to the skin immediately over the seat of the pain; also by the relief more effectually obtained by remedies which have an express influence upon the blood vessels, being employed together with purgatives, than by purgatives alone; for instance, by several doses of calomel, given in succession, at short intervals, and followed by senna or jalap, than by senna or jalap alone.

"Further, a spontaneous vomiting will sometimes bring away from the stomach nothing but a turbid water, and a spontaneous purging will sometimes bring from the bowels nothing but a fluid, which is thin, pale, and inodorous. Hence no relief follows. This striving of Nature is premature and ineffectual.

"Moreover, emetics and purgatives will procure sometimes mere watery evacuations, upwards and downwards, and thus fail altogether of their curative effect.

"The fact seems to be, that the vessels must first pour out the morbid colluvies into the stomach, before it can be rejected out of the body. This its separation from the blood vessels is the first and principal curative effort; its expulsion by vomiting or purging is secondary and consequential, and curative only in a less degree; but still necessary. Emetics and purgatives, so administered, as simply to procure its rejection out of the body, do good, in assisting the last process of the cure; but emetics and purgatives, so administered, as first to aid its separation from the blood vessels, and then its rejection from the body, conspire with Nature in every purpose she endeavours to effect, from the first to the last.

"It appears, therefore, most probable, both from the course of the symptoms themselves, and from the efforts of Nature for her own relief, from the remedies, and from the conditions of their successful operation, that the disorder of the stomach, described as incident to the early stage of fever, is caused and maintained by a morbid action of a peculiar kind, in which its blood vessels are engaged at the time." 133.

In a few words, the fever required evacuations at the beginning, and a judicious administration of tonics pretty early in the disease. Bark and acid were the tonics which proved most serviceable.

Thus, then, we have given our readers a brief account of the complaints prevalent at the Penitentiary—an account which we hope will induce many of them to peruse the original document whence our abstract is derived. It has been seen that the diseases were, a scurvy, a bowel-complaint, a disorder of the brain and nervous system, and a fever. All four, as Dr. L. justly observes, from their extent and frequency, had a claim to be considered "*the disorders*" of the prison. This belief was further strengthened, in respect to two of them, (the flux and the disorder of the nervous system) by the consideration that they were both amenable to one and the same remedy—and intractable by any other.

"What then (it may be asked) was the essence of the disease, considered as a whole? Of the inceptive error, or primary morbid action, from which it arose, I am entirely ignorant; whether it belonged to the blood vessels, or the nerves, or to what particular organ, or system of organs. I only know that its first cognizable effects were seated in the blood vessels, and that they consisted both in the admission of blood into their capillaries, beyond the natural sphere of the circulation, and in its transmission through their capillaries out of the sphere of the circulation altogether; that the consequences were ecchymosis of the skin, and ecchymosis and vascular patches of the mucous membrane of the stomach and intestines, and determinations of blood to the brain and its membranes, and (probably) to the spinal marrow; and that, out of these, arose diseases which obtained different names, according to the manner of their occurrence and the parts upon which they fell, such as the scurvy, and the various species of flux, and the various species of nervous disorder, and some forms of fever, all of which were nevertheless still the same disease, in the conditions of their production." 139.

Dr. L. is well aware that the essence of the disease must have existed prior to these effects—that is, the *causes* of these effects must have pre-existed. But the present state of our knowledge does not enable us to ascertain what that primary link in the chain of morbid action was.

The sixth chapter contains many interesting remarks on what our author terms "intercurrent diseases"—the natural alliance of which to the reigning disorders was problematical. We refer the reader to the work itself for particulars respecting this class of diseases.

Chap. VII. So far, we have been on the subjects of symptomatology, pathology, and therapeutics—we now come to the more difficult topic of etiology. When badgered by the House of Commons respecting the nature and causes of the Penitentiary endemic, the physicians must often have aspirated, in their own minds, the emphatic line of the Roman poet:—

Felix qui potuit rerum cognoscere causas!

But Nature seems exceedingly cautious how she reveals to mortal eyes the *causes* of things, though she makes no secret of their *effects*. We see the flux and reflux of the tide, and we say it is caused by *attraction*, which is merely a term that designates an *effect*. We know nothing of the cause, the nature, or the modus operandi of this said attraction. So of an epidemic or an endemic. We say it is caused by an aerial change or a terrestrial exhalation. But of the nature and causes of these atmospherical constitutions and morbific miasmata we know little or nothing.

On presenting certain tabular views of the state of sickness in the Penitentiary, on the 4th July, 1823, the physicians handed in a report, of which we shall take some notice in this place.

They set out by remarking that, in consequence of distrusting the *viva voce* statements of the prisoners themselves, they had been unable to trace back (on first enquiries) the diarrhœa or scurvy, farther than the preceding Christmas. But greater experience of the conduct of the prisoners induced the physicians to give greater credit to their asseverations, and it was now beyond a doubt that the bowel complaint and scurvy had existed from an early period of the autumn. Even in July, a fortnight after the diet was changed, the diarrhœa began to shew itself. In their report of 5th of April, they ascribed the forms of the disease then prevalent to the influence of diet and cold—but it so happened that many prisoners, admitted since the diet had been improved and the weather had become warm, suffered attacks of dysentery, as also some of the officers of the establishment most employed about the sick. This led them to the conclusion "that some injurious influence had been in operation, over and above the causes to which the epidemic was originally imputed." This could only (in all probability) be one or more of the three following causes—a local malaria—contagion—or something in the moral or physical condition of the people confined within the walls of the Penitentiary. "Whatever it be, say they, it has hitherto eluded our detection—and whether it is, or is not in operation at present, we cannot tell." 158.

The object of this report was to intimate to the committee that the disorder, *as a flux*, had been of longer standing in the prison than they were at first led to believe—that its origin could not be exclusively owing to the diet and the inclemency of the season—that there was some other morbific agency in operation, the nature of which had not been discovered, and was perhaps undiscoverable—that although the extent of the disease was greatly diminished, yet that the prisoners were in a state of health which, to say the least, was dangerously valitudinary—that a return of the disease, in some shape or other, was still to be dreaded. Under these circumstances of doubt, fear, and responsibility, the physicians made application for further assistance, and Drs. Hue, Macmichael, Chambers, and Southey, were appointed by the College for that purpose. Dr. Chambers was prevented from attending by other professional engagements.

Chap. VIII. *History continued.* Without affixing any stigma of malaria on the Penitentiary, Drs. Latham and Roget wisely concluded that change of air and residence would be salutary to the mass of prisoners, as it is well known to be to individuals in private life, who have long laboured under illnesses, and are subject to relapses. Accordingly, all the prisoners were eventually removed from Millbank to situations deemed more eligible for the recovery of their health. One hundred and twenty were sent to the Ci-devant Ophthalmic Depôt, in the Regent's Park—200 to the Ethalion Hulk, at Woolwich.

"The benefit of change of air and situation was immediately apparent, both upon the women at the Regent's Park, and the men at Woolwich. In a fortnight after their removal, there was much less complaint of illness among them, and their general aspect bore the appearance of returning health." 167.

Still about 400 remained in the Penitentiary. Their health remained nearly stationary, but they were harassed occasionally with their old complaints, especially the diarrhœa, though not in so frightful a form as previously. "But if the disease, even now, went beyond a certain degree, it was in vain to attempt its abatement by any medicine but mercury—a fact to which the physicians, lately called in, bore ample testimony with Dr. Roget and myself." 168.

On comparison, during the month of September, the balance of health was found to be greatly in favour of the prisoners removed to Regent's Park and Woolwich, although only six weeks previously their constitutions were in a much worse state than those of the prisoners left in the Penitentiary. Another month rolled away, and yet "the prisoners at Millbank, although they were suffering no formidable disease, still experienced some insurmountable impediment to the recovery of their health." In this respect, their condition exhibited a melancholy contrast with that of the prisoners removed to the places beforementioned, especially Woolwich. This state of things led to the removal of all the prisoners to hulks fitted for their reception at Woolwich. Early in December, the Penitentiary was destitute of its wretched inmates. The prisoners removed to Regent's Park did not recover nor retain their health near so fast nor so firmly as those in the hulks at Woolwich.

"Immediately after their removal, both alike seemed to throw off the remaining symptoms of their disease, and to put on the appearance of returning health; yet both afterwards continued alike to suffer an occasional recurrence of their disease, chiefly in the form of diarrhœa. There was, however, this manifest difference between the two, that while those on board the hulks at Woolwich continued to recover their general health, in spite of frequent occasional attacks of diarrhœa, the females at the Regent's Park did not, in respect of their general health, go on to improve during more than the few first weeks after their removal. Moreover, among the prisoners on board the hulks, during a period of between four and five months, there had not occurred a single case of formidable disease; whereas, among those at the Regent's Park, during the same period, there had been several. At the end of November, several women at the Regent's Park were seized, nearly at the same time, with those insidious forms of phrenitis which, five months before, had been a great cause of alarm at the Penitentiary, and had, in some instances, proved fatal. Dr. Roget, to whose care the hospital in the Regent's Park was especially assigned, found himself compelled to resort to the same remedy by which these symptoms were formerly brought under control, and with the same success." 175.

On the 21st of January, 1824, the prisoners were removed from Regent's Park to Woolwich. The whole now amounted to 635 in number—and the medical superintendance was placed in the hands of practitioners on the spot. Mr. Bayles had the care of the men, and Mr. Pratt, apothecary to the Penitentiary, that of the females. The physicians, however, continued to visit the hulks at intervals. It was during these occasional visitations that the following curious facts were observed and noted by the physicians.

The prisoners transported to the hulks, though at different periods of the year, all experienced a striking and almost instantaneous change for the better. In ten days after removal they were, in general, free from complaint. This speedy and surprising amendment was very encouraging, but, in every instance, it was fallacious; for, after a temporary pause, the disorder returned in the form of diarrhœa. It was, however, mild in its symptoms, though evidently the same disease that had prevailed at the Penitentiary. Another remarkable circumstance was, that neither the total absence of the disorder during a considerable period, nor the complete re-establishment of the general health, furnished any security against the recurrence of the flux. This liability to the disorder rendered the state of the prisoners very fluctuating and uncertain—consequently, it was impossible to anticipate what would be their condition from one week to another. These relapses could not be traced to any external causes, but could only be rationally attributed to the state of the bowels themselves, which were ever prone to take on the same disordered function which had formerly caused so much trouble and even fatality. In no case of relapse, at Woolwich, did the patient die, and therefore there was no opportunity for post-mortem investigations. Several dissections, however, performed at other times, and with different views, seemed to throw some light, collaterally on the question respecting the actual state of the intestines in those so subject to recurrence of the diarrhœa. In those who died at the Penitentiary, after symptoms immediately referrible to the head, having formerly suffered disorder of the bowels, but being a long time free from it, the physicians found ulcers of the intestines, which were otherwise exempt from disease. These ulcers were few in number, not more than three or four throughout the whole tract of the bowels. They were of very small extent, and in progress towards reparation. A young man, who had originally suffered the bowel-complaint very severely at Millbank, and whose life had been rescued by the mercurial treatment, had occasional relapses at Woolwich, notwithstanding the re-establishment of his general health. He was ultimately pardoned and set at liberty. A short time afterwards, he presented himself to Dr. Latham among other patients applying for admission into the Middlesex Hospital. He was then a miserable object, worn down and emaciated by impoverishing diet, and the hardships he had suffered since his liberation. His complaint was diarrhœa, with severe pain in the head. The diarrhœa was soon arrested; but the disorder of the head assumed the form of that insidious phrenitis already described, and he died. On dissection, marks of inflammation and effusion were found in the head. "In the intestines, after careful examination, nothing was found but three minute spots, exhibiting the appearance, already described, of ulcers in the process of reparation." In another case, that of a young woman who had suffered the flux at the Penitentiary, with several slight relapses, and who died, in the course of the last summer, at the Westminster Hospital, of phthisis; healing ulcers were found in the intestines, besides the usual disease of the lungs. They were very circumscribed in their situation, and entirely free from surrounding inflammation. We cannot but agree with our most intelligent author in the following conjectures.

"It is not unreasonable to conjecture, that the same condition of the bowels, which was found in these cases, existed also in those who suffered the frequent recurrence of diarrhœa at the hulks. A few minute ulcers, somewhere in the tract of the intestines, tardily undergoing the process of reparation, and without any surrounding inflammation, constitute just that sort of morbid condition which is calculated to produce such a disorder. Being very circumscribed in extent and free from inflammation, they would be capable of existing without constant injury to the general health, and even without constant impediment to the functions of the parts to which they belonged; yet, from the natural irritability of those parts, they would still be liable to produce temporary disorders, under the influence of occasional causes; and such disorders would continue apt to recur, until the process of their tardy reparation was complete." 183.

The therapeutical indications were grounded on this view of the case. In the milder forms of the disease they abstained from mercury, and had recourse to opiates, absorbents, and aromatics, with success. But, in some instances, these failed, and then the physicians were forced to have recourse to the mercurial treatment already detailed.

"Such a disorder arose on board the Narcissus; and the symptoms seemed to bespeak a morbid action of an acute kind, set up afresh, or ingrafted upon the old. The women on board the Narcissus had, since their arrival at Woolwich, in November, rapidly recovered their general health, and had been strikingly exempt from their former complaints, when, in the month of March, there were found among them numerous instances of disorder, expressly referable to the stomach. It was of a severe and formidable kind, and did not strictly resemble any of those forms of disorder which have been described. There was a sense of sinking, with extreme pain over the whole epigastric region, great impatience of pressure, and enormous distention of the abdomen, violent retching, with the rejection, in several cases, of pure florid blood. The blood was generally small in quantity, but, in a few instances, it amounted to more than a pint. The pulse was feeble. Neither food nor medicine would remain upon the stomach; both were rejected, with extreme aggravation of all the symptoms.

"The disorder had already existed during several days, and various methods of treatment had been employed, when Dr. Roget and myself were sent for to Woolwich. We found ten women at least in a state of peril. The remedies used had produced no abatement of the symptoms, which were daily becoming worse in every case. Mercury had not been of the number, the apothecary conceiving that he acted in conformity with our wishes, when he scrupulously withheld it.

"Now, although the order of symptoms which we now witnessed was not precisely identical with any we had formerly seen at the Penitentiary, yet it was impossible not to regard them as partaking of the same nature; and surely, upon the failure of every other remedy, we were not justified in withholding that, which had already rescued life under various circumstances of peril belonging to kindred diseases. Accordingly, we resorted to calomel and opium. Five grains of calomel and one grain of opium were first administered to several patients; but there followed, to our disappointment, an aggravation of all the symptoms. The retching, and vomiting, and spasmodic pain were prolonged through the night. Yet it was not the remedy itself, but the largeness of the dose, that produced the disagreement; for when one grain, or two grains of calomel, and a fourth, or half of a grain, of opium, were given every second, or every third hour, they were retained, and the stomach was tranquillized, and there followed a gradual abatement of the symptoms. Still all the symptoms, and especially the pain, were not finally removed, until the gums were perceptibly sore.

"This affection of the stomach was different from that already described as the concomitant of fever. It did not tend to a critical termination, and its relief, although it was procured by the same remedy, was brought about in a different way; not by its producing a discharge of morbid secretions, but (as far as we could judge) by its specific influence upon the constitution." 187.

In the course of ten days, this form of disease attacked nearly half the women, on board of the Narcissus, in succession, and there was a continual apprehension that it would prove fatal to some. In the mean time, almost all on board the same vessel, who were not afflicted after this manner, suffered either some form of the bowel complaint, or some affection of the brain or nervous system, such as formerly prevailed at the Penitentiary. Hardly any escaped severe illness; but none died. There was no one peculiar cause, of a physical nature, which could be assigned for these late attacks; but there were many causes connected with their condition as prisoners. Speaking of the females removed from Regent's Park to the Heroine Hulk at Woolwich, our able and intelligent author observes:—

"Deplorable, indeed, was the condition of all these wretched women, both on board the Narcissus and the Heroine, in respect of their bodily sufferings, but more deplorable still (if possible) in respect of their mental misery. It was painful to witness the manner in which they all abandoned themselves to despair, and those especially who had still before them the prospect of a long captivity. Every day brought some fresh proof how great was the influence of mental distress in augmenting bodily pain and sickness. Whatever circumstances were calculated to make a strong impression upon the spirits, threw them back at once, from a state of convalescence, into absolute disease. I will mention one such circumstance, as an example.

"Upon the recommendation of the committee, individuals were pardoned, from time to time, for good conduct. Recently, claims for such recommendation had been more easily admitted, and, consequently, pardons had become very numerous. Hence, the prisoners themselves naturally began to think that the claims of all were nearly equal, and every one pleased herself with believing that she should be the next who would be set at liberty. Whenever, therefore, an individual was pardoned, all the rest were thrown into an agony of the bitterest disappointment, and were, at the same time, overtaken by disease. It was not a mere nervous or hysterical ailment, but some actual form of real disease, such as they had before suffered, and requiring the strictest medical treatment for its relief." 192.

Although much accurate information, concerning those who were liberated, could not be obtained, yet it appeared, from several who were afterwards seen, that their state of health was greatly improved. Under these circumstances, the physicians and Home Secretary, Mr. Peel, humanely came to the conclusion, that a general gaol delivery was the best thing that could be done, conceiving, most justly, that "the course of their sufferings, during more than twelve months, might be thought fully equivalent to many years' imprisonment." The committee, therefore, corresponded with their friends in various parts of the kingdom, so as to secure an asylum for these unfortunate females, and then they were all returned to their native homes. By an Act of Parliament, obtained on the 12th April, 1824, the male prisoners, 400 in number, were distributed among the hulks at Sheerness, Chatham, and Woolwich, subject to the discipline and labours of other convicts, and from thence, the accounts of their health have been most satisfactory. Thus ends this "strange eventful history."

Chap. IX. *Etiology*. It will be remembered by our readers that the physicians originally considered the Penitentiary endemic as arising *exclusively* from diet and atmospheric inclemency, but that farther observation induced them to alter their opinion. In their July report, they intimated to the committee, that there was some agency in operation over and above that originally assigned—that, in fact, there was suspicion of contagion and local malaria.

Dr. Latham now comes to the facts and circumstances that bear on these two points, with the view of enabling his professional brethren to form their own opinion concerning them—keeping the topics of contagion and malaria separate.

*First.—Contagion.* At the date of their report, in April, 1823, the disease had been found exclusively among the prisoners. Subsequently, however, several officers of the establishment, especially those most employed about the sick, became affected. Eighteen, in all, of this class, suffered from the disease, among whom were the chaplain and some of his family. Prisoners, too, who were admitted subsequent to the bettering of the diet and inclemency of the winter, were not exempt from the disease. After the removal of the prisoners to Woolwich and the Regent's Park, certain circumstances also occurred, which still farther strengthened the idea of contagion. Thus, three persons employed at the Ophthalmic Hospital, and who had not been at the Penitentiary, suffered disorder of the bowels, under one of the forms of cholera, which the complaint assumed at Millbank. "But it was at a time of the year when cholera morbus was every where a frequent complaint." Every one must perceive that this event proves nothing at all; and, therefore, it is not necessary to waste a moment's consideration on it. Neither do we think the circumstance of a few of the officers being affected with bowel complaint in the Penitentiary, goes any length in affixing a contagious character on the disease. The officers of the institution, residing, as they did, under the same roofs, or, at all events, within the same walls, must have been exposed to the same causes, (if these were of a local or malarious nature) as the prisoners, though their moral and physical condition would enable them to resist the operation of the morbific agent much longer and much more generally than the prisoners.

The next fact brought forward by our author is this. The female prisoners on board the Narcissus had been convalescent during several weeks, when the females of the Regent's Park (of whom several had suffered recently from renewed attacks of the disorder) were removed to Woolwich, and put on board the Heroine. Both vessels were moored close together, and some women from the Heroine were transferred to the Narcissus. "Not many days afterwards, there was a general complaint of illness on board the latter vessel, which terminated in that form of the disease which has been described as occurring in the month of March, and thence it was concluded that the disease was communicated to the prisoners of the Narcissus." This conclusion was too absurd to be entertained by our intelligent author, with whose sentiments, as contained in the following passage, we entirely agree.

"Now, if this were the fact, the disease was not only contagious, but contagious in a manner peculiar to itself. For it has never been shewn (as far as I am informed,) that the convalescents from any disease are capable of re-infection by those who are the more recent subjects of it.

"I am not disposed, therefore, to believe that the prisoners of the Narcissus, in this instance of their sudden disorder, were re-infected by those of the Heroine, because the fact itself would be contrary to general experience. And, further, I am not disposed to believe it, because transitions from a state of convalescence to a state of disorder, had been just as sudden among the men on board the Dromedary and the Ethalion, when there was no possibility that it could have been derived from contagion, unless you suppose the same people capable of receiving and communicating the same complaint, from and to each other, indefinitely." 208.

The last fact adduced is that of a waterman, who attended on the Narcissus, becoming affected with a severe attack of dysentery. Dr. Latham's own remark on this solitary instance is quite in point. "The single instance of the waterman, surely, cannot justify this conclusion"—of contagion. Certainly not; we may therefore consider the question of contagion, as far as this endemic is concerned, as disposed of. Not that we mean to deny that such a disease as that which harrassed the Penitentiary prisoners might be, or rather might become, contagious. We think the thing by no means impossible. All we mean to insist upon, in the present instance, is, that such fact or occurrence is not proved, nor even rendered probable by the historical documents of the Penitentiary endemic.

We next come to the important question of local morbific agency—or in other words, malaria.

The very able report drawn up by the physicians on the 11th of October, 1823, contains the sum and substance of facts and circumstances bearing on the above question, and we wish we could spare space for this interesting document; but we cannot, in consequence of having already far exceeded our bounds. In this report, mention is made of certain diaries, or day books, kept by the apothecary of the Institution, but which, for reasons unknown, were never produced till a few weeks before the date of this report, when Mr. Pratt voluntarily produced them, with this remarkable expression, that "it was fortunate he had preserved them." By these books it was proved, that bowel complaints had predominated among the prisoners for years,—indeed from the first institution of the Penitentiary.

"The following Table gives the number of prisoners in the Penitentiary every year since the year 1816, with the number of cases treated as diarrhœa in every year:—

| — | *1816. | 1817. | 1818. | 1819. | 1820. | 1821. | 1822. |
|---|---|---|---|---|---|---|---|
| Prisoners - - | 72 | 212 | 246 | 351 | 609 | 798 | 866 |
| Cases of diarrhœa - | 23 | 104 | 106 | 82 | 85 | 87 | 88 |

"We are aware that inferences concerning the nature of a disease deduced from the remedies employed for its cure would, in general, be hasty and inconclusive. But, as in the present case, the nature of the

* "The numbers in this year refer to a period of six months only."

disease is unequivocally indicated by the particular remedies used, we may, with certainty, conclude that diarrhœa has existed in the Penitentiary from its first establishment, and that it has prevailed in various degrees of extent at different periods; that, proportionably to the number of prisoners, it prevailed to the greatest degree during the year immediately after its establishment, and that it has prevailed in a less and less degree each succeeding year, down to the period when the present epidemic was discovered." 214.

The above discovery was made by the quantities of chalk mixture and tincture of opium prescribed—a medicine which, it is needless to observe, could not have been prescribed for any other complaint in the whole range of nosology. Long before the production of the books by Mr. Pratt, the physicians had become morally convinced that "complaints which had flux of the bowels for their prominent symptom, had been frequent in the Penitentiary during former years." The suspicion was first, we believe, engendered in Dr. Macmichael's mind, in consequence of a letter written by Mr. Pratt, in March 1822, and produced before the Committee in 1823. It was written when a change of diet was contemplated at the Penitentiary, and contained the following remarkable expression :—"At the same time, I am fearful that a long continuance of soup, however strong, might produce affections of the bowels."*

"This warning was not forgotten when the evil became manifest. Mr. Pratt was acknowledged by all to have predicted truly, and allowed considerable credit. He was himself accustomed to refer us to this prophecy and its verification, not without some exultation ; and indeed, well he might ; for if, without any help or suggestion from what had already occurred, he really foresaw, not that some disease or other, but expressly the very disease would follow the change of diet, which actually did follow, it is one of the most splendid instances of medical anticipation upon record. Nevertheless, we were slow to allow him more than the credit due to a sensible man, rightly conjecturing what would take place hereafter, from what had heretofore fallen under his own observation. In short, we were quite certain, that the prophecy could have had no other foundation than in the *bonâ fide* experience of the prophet." 222.

Still the physicians had no positive proofs of their suspicions, till Mr. Pratt produced the books which he had "fortunately kept," "from which it appeared, by the testimony of Mr. Pratt's own hand-writing, that at the very time he was prophesying to the Committee, that certain diseases would take place upon

---

* See Report of the Penitentiary, p. 101, published by order of the House of Commons, in July, 1823.

their projected change of diet, he had already been prescribing most largely for those very diseases ever since the foundation of the prison, for six years in succession." 222.

One thing will strike our readers with surprise, and that is, that Mr. Pratt *alone* should be acquainted with the facts abovementioned.

"His books, however, soon cleared up this perplexity. For in perusing them we found, that the apothecary alone was *fully* acquainted with the facts upon which the anticipation was grounded, and therefore that he alone could confidently entertain it. The prisoners, for whom chalk mixture, *&c.*, was so largely employed were, for the most part, under Mr. Pratt's exclusive care. More than half the number had medicine given to them while they were following their ordinary occupations in the prison. The functions of the medical superintendent and the apothecary were so far divided, that while the daily business of the former was only with the prisoners in the infirmaries, in prescribing for their severer maladies, that of the latter was moreover with the prisoners in the Penitentiary at large, in prescribing for various ailments which did not require their removal into the infirmaries." 225.

One other circumstance, bearing on the question of malaria, deserves to be noticed. It has already been shewn that an affection of the brain and nervous system formed as much a part of the Penitentiary Endemic, as did the bowel complaints themselves, consisting chiefly of head-aches and vertiginous sensations, in many instances aggravated into tremors, convulsions, and phrenzy.

"We needed nothing but the record before us of the medicines prescribed, to ascertain the prevalence of bowel complaints in the Penitentiary since its foundation; but we were indebted to the *voluntary* suggestion of Mr. Pratt, for enabling us to trace out retrospectively head-ach and vertigo through every page of his own books. So confident was he, from a knowledge of his own methods of prescribing, that certain remedies there recorded were given by him for headach and vertigo, that he undertook to draw up, and actually did draw up, a list of the numbers afflicted with these disorders from the establishment of the Penitentiary to the present time. These numbers were almost as great as of those who suffered the bowel complaints in every year. They were arranged in a tabular form, and were to have made part of the Report presented to the committee. But, upon deliberation, it was thought better not to offer any thing to the committee which was not self-evident, or which stood in need of explanation beyond the mere statement of the fact." 227.

These circumstances, taken separately and collectively, leave no doubt in our minds, that a disorder of the same general character with the late Endemic, has prevailed in the prison since its establishment, and that its tremendous development at last, was owing to the impoverishing diet, the inclemency of season—and perhaps other auxiliary or exciting causes totally unknown to us at present. We think the admission of the local malaria also, must still farther weaken the probability (small as that probability was before) of the influence of contagion in originating or propagating the Endemic in question. This appears to be the impression on the mind of Dr. Latham himself.

We cannot dismiss this subject without adverting, though unwillingly, to the conduct of the apothecary. We have nothing to do with motives, and always wish to consider them in a point of view favourable to the individuals who harboured them. It is only from *actions* we judge—but we claim the privilege of drawing inferences from actions, as well as of awarding to actions their meed of censure or approbation.

We think that no unprejudiced or reflecting mind will give Mr. Pratt credit for *divination* in foretelling the prevalence of bowel complaints among the prisoners. That it was an *ex post facto* prophecy no man can doubt. Why did not Mr. Pratt then, candidly and manfully state the fact of the prevalence of bowel complaints in the prison, at an early period of the Endemic, and thus, by leading to an early removal of the prisoners, prevent an immense sum total of disease and death ?—The disclosures made in the work before us render it pre-eminently imperative on Mr. Pratt to answer this question, for the sake of his own character.

The remainder of this masterly performance, is occupied with a defence which has been imposed upon our author by a most unjustifiable attack on the reports of the physicians, made by an extra-professional gentleman, Mr. Holford, one of the Committee. Although it was perhaps proper and necessary for Dr. Latham, situated as he was, to enter into a controversy with Mr. Holford, it is quite unnecessary for us to lay any part of the dispute before our readers. Mr. Holford, like many other men, being perfectly ignorant of any of the principles of medical science, conceives himself an infallible judge upon all points and questions, however intricate and difficult! Being determined to make out the Penitentiary a perfect Paradise, in respect to salubrity, as he had before attempted to render ox-head soup a fare of ambrosia and nectar for its inmates, he has, in the teeth of facts, undertaken to prove that no bowel complaints were prevalent in the Penitentiary, prior to the late extension of it into a disastrous endemic. And how does this sapient sage make out his argument? Because, when Mr. Pratt was issuing forth his daily and nightly rations of chalk-mixture and laudanum to the prisoners, for years before, he did not stand High Priest in the Temple of Cloacina, and measure, with ounce, drachm, and minim glasses, the votive offerings of the Fair Penitents! *Ergo*, the bowel complaints were all feigned, in the Penitentiary—and Millbank is the Montpellier of Middlesex. Q. E.D.*

With such quibbling and drivelling we have no patience; and we only wonder how Dr. Latham could have adhered to such mild language while answering the insulting taunts and illiberal insinuations thrown out in the report of Mr. Holford.

We have offered our readers sufficient samples of the work now reviewed, to form a judgment of its merits. For our own parts, we have no hesitation in asserting that it is one of the ablest and most interesting works which we have perused for some years. It will stamp its author as a man of sterling talent, sound judgment, and accurate observation, as long as medicine is cultivated as a science.

